# Salvianolic Acid A Ameliorates Arsenic Trioxide-Induced Cardiotoxicity Through Decreasing Cardiac Mitochondrial Injury and Promotes Its Anticancer Activity

**DOI:** 10.3389/fphar.2018.00487

**Published:** 2018-05-09

**Authors:** Jing-yi Zhang, Min Wang, Rui-ying Wang, Xiao Sun, Yu-yang Du, Jing-xue Ye, Gui-bo Sun, Xiao-bo Sun

**Affiliations:** ^1^Institute of Medicinal Plant Development, Peking Union Medical College and Chinese Academy of Medical Sciences, Beijing, China; ^2^Beijing Key Laboratory of Innovative Drug Discovery of Traditional Chinese Medicine (Natural Medicine) and Translational Medicine, Beijing, China; ^3^Key Laboratory of Bioactive Substances and Resource Utilization of Chinese Herbal Medicine, Ministry of Education, Beijing, China; ^4^Zhongguancun Open Laboratory of the Research and Development of Natural Medicine and Health Products, Beijing, China

**Keywords:** salvianolic acid A, arsenic trioxide, cardiotoxicity, mitochondria, mitochondrial biogenesis

## Abstract

Arsenic trioxide (ATO) is used as a therapeutic agent in the treatment of acute promyelocytic leukemia (APL). The therapeutic use of arsenic is limited due to its severe cardiovascular side effects. The cardio-protective effect of salvianolic acid A (Sal A) against ATO cardiotoxicity has been reported. However, the distinct role of the mitochondria in the cardio-protection of Sal A is not understood. The aim of this study was to determine whether Sal A preconditioning protects against ATO-induced heart injury by maintaining cardiac mitochondrial function and biogenesis. For the *in vivo* study, BALB/c mice were treated with ATO and/or Sal A. For the *in vitro* study, we determined the effects of ATO and/or Sal A in H9c2 cardiomyocytes. Our results showed that ATO induced mitochondrial structural damage, abnormal mitochondrial permeability transition pore (mPTP) opening, overproduction of mitochondrial reactive oxygen species (ROS), and decreased the ATP content. Sal A pretreatment alleviated the ATO-induced mitochondrial structural and functional damage. In this study, ATO decreased the expression level of the peroxisome proliferator activator receptor gamma-coactivator 1 (PGC-1α) and disrupted the normal division and fusion of mitochondria. Sal A pretreatment improved the dynamic balance of the damaged mitochondrial biogenesis. Moreover, the combination treatment of Sal A and ATO significantly enhanced the ATO-induced cytotoxicity of SGC7901, HepaRG, K562 and HL60 cells *in vitro*. These results indicated that Sal A protects the heart from ATO-induced injury, which correlates with the modulation of mitochondrial function, and the maintenance of normal mitochondrial biogenesis.

## Introduction

Arsenic trioxide (ATO) is a widely recognized antileukemic drug used for the treatment of newly diagnosed and relapsed acute promyelocytic leukemia (APL) (Kwong and Todd, 1997). However, its chemotherapeutic usage has caused side effects such as cardiac arrhythmia, ventricular tachycardia, or even sudden cardiac death (Zhang et al., 2013). The heart is a highly energy-consuming organ. To maintain normal contractile coupling, cardiomyocytes rely on a continuous oxygen supply and oxidized substrates to produce enough ATP to meet their actual energy requirements (Arany et al., 2005). Mitochondria are important sites for the cellular synthesis of ATP, and the energy generated is used to maintain the normal physiological functions of cells and for participation in a variety of cellular activities such as intracellular Ca^2+^ regulation and metabolism (Palomer et al., 2013). During the course of myocardial injury, changes in the structure and function of the mitochondria result in the destruction of the mitochondrial homeostatic balance, resulting in increased mitochondrial-derived ROS production, Ca^2+^ overload, increased mitochondrial inner and outer membrane permeability, and changes in mitochondrial morphology (Sedlic et al., 2010; Wang et al., 2015). These events interact with each other and play an important role in regulating the metabolic functions of cardiomyocytes and in cell death (Bernardi and Rasola, 2007).

Mitochondria are highly dynamic organelles that maintain the homeostasis of the mitochondrial network through continuous fission/fusion to adapt to the different energy and functional needs of the cell (Luz et al., 2017). Mitochondrial biogenesis refers to the growth and differentiation of mitochondrial precursors (Nirwane and Majumdar, 2017). Its fusion and cleavage properties ensure the correctness of mitochondrial network structure for mitochondrial biogenesis (Bach et al., 2003; Garnier et al., 2005). PGC-1 is an important regulator of mitochondrial oxidative metabolism and biogenesis (Di et al., 2018). An energy deficiency and the decreased expression of PGC-1 transcription and activity are hallmarks of cardiac dysfunction and further lead to decreased energy metabolism, thereby contributing to systolic heart failure (Kertai et al., 2005). The proteins involved in mitochondrial division in mammalian cells mainly include Drp1 (dynamin-related protein-1) and its receptor proteins (FIS1, MFF). The proteins involved in fusion mainly include the outer membrane fusion proteins MFN1 (mitofusin1) and MFN2 (mitofusin2) and the inner membrane fusion protein OPAl (opticatrophy 1) (Givvimani et al., 2015; Prakash and Kumar, 2016).

In recent years, the role of mitochondria in the process of myocardial injury has received widespread attention. It has been reported that mitochondrial dysfunction plays an important role in the initiation of heart diseases such as coronary heart disease and heart failure (HF) (Murakami et al., 1999; Makazan et al., 2007). Emerging evidence has showed that mitochondrial damage induced by ischemia/ reperfusion (I/R) injury impaired the mitochondrial function. Conversely, it further contributes to mitochondrial oxidative damage and ultimately promotes the opening of mPTPs and thereby induces mitochondrial-dependent apoptosis (Sedlic et al., 2010; Luo et al., 2013). Damaged mitochondrial ultrastructure and mitochondrial dysfunction were involved in HF induced by various causes. Reduce the damage of mitochondria, improve mitochondrial oxidative phosphorylation function is the key treatment for HF (Li et al., 2016). Therefore, protecting the structure and function of the mitochondria and maintaining mitochondrial biogenesis may be an important strategy for the prevention and treatment of ATO cardiotoxicity.

**FIGURE 1:**
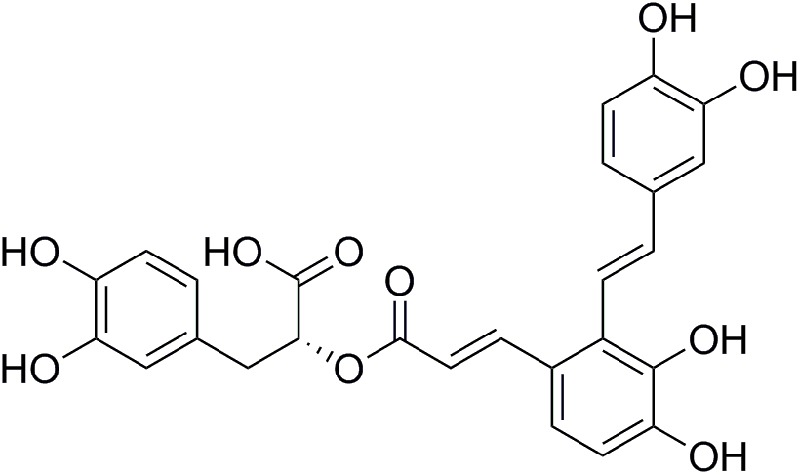
The molecular structure of Sal A.

*Salviae miltiorrhizae* (also known as Danshen) is one of the most frequently used Chinese herbs and is believed to have effects on cardiovascular diseases (Yan et al., 2015). Salvianolic acids have been found to have potent antioxidative capabilities due to their polyphenolic structure (Wang et al., 2012). Salvianolic acid A (Sal A, **Figure 1**) is the most potent antioxidant of the salvianolic acids (Zhang et al., 2011). Our previous studies showed that Sal A enhances antioxidant enzyme activity, decreases ROS overproduction, and attenuates ATO-induced cardiac injury in H9c2 cells (Zhang et al., 2017). Moreover, it has also been reported that Sal A attenuates myocardial ischemia/reperfusion injury by preserving mitochondrial function, improving the energy and antioxidant state (Li et al., 2017). These results indicate that the mitochondria may be a potential therapeutic target of Sal A to reduce ATO-induced cardiotoxicity.

Therefore, the major purpose of this study is to investigate the effect of ATO on mitochondrial dysfunction and mitochondrial biogenesis, and whether Sal A could antagonizing the cardiotoxicity of ATO by preventing the mitochondrial injury without changing the anticancer activity of ATO. In this study, we examined the combination treatment of ATO and Sal A on the cardiotoxicoty and then examined the combination treatment *in vitro* on SGC7901, HepaRG, K562 and HL60 cells. The results showed that Sal A eliminated the cardiotoxicoty of ATO and enhanced ATO anticancer activities *in vitro*.

## Materials and Methods

### Ethics Statement

All animal experiments were approved by the Medical Ethics Committee of Peking Union Medical College and were in accordance with the national institutes of health regulations for the care and use of animals. All efforts were made to minimize suffering.

### Materials

Sal A (content ≥ 98%) was purchased from Shanghai Winherb Medical S&T Development Co., Ltd. (Shanghai, China). ATO was obtained from Harbin YI-DA Pharmaceutical Ltd. (Harbin, China). All the anti-bodies were purchased from Abcam (Cambridge, England). All chemicals were acquired from Sigma (St. Louis, MO, United States).

### Cell Culture and Treatment

H9c2 rat ventricular cardiomyocytes, SGC7901, HepaRG, K562 and HL60 cells were purchased from the Cell Bank of the Chinese Academy of Sciences (Shanghai, China) and cultured as previously described (Sun et al., 2011). The cells were grown after reaching 85% confluence. The H9c2 cells were divided into following groups: (1) Control group (Control); (2) ATO group (ATO-2.5/ATO-5/ ATO-10): H9c2 cells were treated with different concentrations (2.5, 5, 10 μM) of ATO for 24 h; (3) Sal A group (Sal A): H9c2 cells were treated with 10 μM of Sal A for 4 h; (4) ATO + Sal A group (ATO + Sal A): H9c2 cells were pretreated with 10 μM of Sal A for 4 h and then treated with ATO (10 μM) for 24 h.

The cancer cells were divided into following groups: (1) control cells; (2) cells treated with ATO (5 μM) for 24 h; (3) cells treated with Sal A alone (25 μM) for 24 h; and (4) cells cotreated with 5 μM ATO and 25 μM Sal A for 24 h. Cell viability was detected using a Cell Counting Kit-8 assay (Dojindo, Kumamoto, Kyushu, Japan) according to the instruction.

### Measurement of Mitochondrial Membrane Potential (Δѱm) via JC-1

JC-1 (Invitrogen, Waltham, MA, United States) was used to determine the mitochondrial transmembrane potential changes as previously reported (Zhang et al., 2017). Different groups of H9c2 cells were seeded in 6-well plates. After the above mentioned treatment, the cells should be centrifuged at 800 rpm for 5 min and the pellet should be incubated with JC-1 (2 μM final concentration) in the dark at 37°C for 30 min. Then the cells were washed three times with PBS and subjected to flow cytometry (FACSCalibur Flow Cytometer, BD Biosciences, CA, United States).

### Generation of Mitochondrial ROS

To explore the mitochondrial ROS levels, the cells were treated with 0–10 μM ATO for 24 h. Then, the cells were harvested and washed with 1× wash buffer. The cells were centrifuged at 800 rpm for 5 min, and the supernatant was discarded. The level of mitochondrial ROS was measured using a MitoSOX^TM^ Red mitochondrial superoxide indicator (Invitrogen, Molecular Probes). After treatment, the cells were resuspended in 300 mL PBS with 5 μM MitoSOX reagent, incubated at 37°C in the dark for 15 min. The cells were then washed three times with PBS, and the mitochondrial ROS levels were analyzed using flow cytometry.

### Determination of ATP Content

Using a commercially available kit (Nanjing Jiancheng Bioengineering Institute, Nanjing, China), ATP content was measured according to the manufacturer’s instructions. Samples were mixed with the reaction solution and bathed at 37°C for 30 min. Precipitant was added at 4000 rpm for 5 min. Then the supernatant was reacted with the coloring solution for 2 min and the terminator was added at room temperature for 5 min. Finally, the absorbance was measured at 636 nm using a microplate reader.

### Animals and Treatments

Male BALB/c mice weighing 18–20 g (Vital River Laboratories, Beijing, China) were used in this study. The procedure reported herein was approved by the local animal committee. The procedures and interventions were approved by the Institutional Animal Use and Care Committee (Registration Number: #IMPLAD2017030715).

#### Part 1

The mice were randomly divided into the following seven groups (*n* = 15/group). (1) Control group (Con) mice were given intraperitoneal (i.p.) injections of normal saline. (2) ATO low-dose group (L) mice were treated with ATO i.p. at a dose of 1 mg/kg for 14 days. (3) ATO middle-dose group (M) mice were treated with ATO i.p. at a dose of 2 mg/kg for 14 days. (4) ATO high-dose group (H) mice were treated with ATO i.p. at a dose of 4 mg/kg for 14 days. (5) ATO short-term group (3) mice were treated with ATO i.p. at a dose of 4 mg/kg for 3 days. (6) ATO middle- term group (7) mice were treated with ATO i.p. at a dose of 4 mg/kg for 7 days. (7) ATO long-term group (14) mice were treated with ATO i.p. at a dose of 4 mg/kg for 14 days.

#### Part 2

Sixty mice were randomly divided into the following four groups (*n* = 15/group). (1) Control group (Con) mice were given intraperitoneal (i.p.) injections of normal saline. (2) Sal A-treated group (Sal A) mice were treated with 3 mg/kg Sal A (i.p.). (3) ATO-treated group (ATO) mice were treated with ATO i.p. at a dose of 4 mg/kg for 14 days. (4) ATO + Sal A group (ATO + Sal A) mice were treated with 3 mg/kg Sal A 1 h before ATO administration. All treatments were administered via tail vein injection for 2 weeks.

### Measurement of Myocardial Enzymes Activities

All the animals were fasting the day before the autopsy. Blood samples were collected via inner canthus using a capillary tube. Serum was separated after the blood samples were centrifuged at 3000 × *g* for 15 min within 1.5 h after collection. Serum myocardial enzyme activities of LDH and AST were measured with corresponding detection kits according to the manufacturer’s instructions (Nanjing Jiancheng Bioengineering, China).

### Identification of Cardiac Mitochondria With Transmission Electron Microscope

The heart tissue was fixed in 2.5% glutaraldehyde in phosphate buffer (0.1 M, pH 7.4) for 2 h. After washing in the same buffer 5 times, the tissue samples were post-fixed in 1% osmium tetroxide (TAAB) in 0.1 M phosphate buffer for 1 h and then dehydrated and embedded in Epon 812 (TAAB) according to a standard procedure (Luo et al., 2017). Ultrathin sections were stained with uranyl acetate and lead citrate and observed under a JEOL JEM1230 transmission electron microscope (JEOL Ltd., Tokyo, Japan).

### Isolation of Cardiac Mitochondria

Mitochondria were extracted using a Mitochondria Isolation Kit (Beyotime Institute of Biotechnology, China) according to the manufacturer’s instructions. The samples and lysate were fully mixed and centrifuge at 800 rpm for 5 min at 4°C. The supernatant was what we need, because nucleus, large membrane fragments, and unlysed cells were at the bottom of the tube. Then the supernatant was gently added to the upper layer of the reaction solution and centrifuged at 800 rpm for 5 min at 4°C. The precipitate was mitochondria, resuspended by addition of a rinsing solution, and centrifuged at 800 rpm for 5 min at 4°C. Resuspend mitochondrial pellets using stock solution and store at -70°C.

### Determination of Mitochondrial Permeability Transition Pore (mPTP) Opening

A mPTP fluorescence detection kit (Genmed Scientifics Inc., Shanghai, China) was used to assess the mPTP opening. Calcein was used to stain the mitochondria. This dye selectively aggregates inside the mitochondria, resulting in green fluorescence. The dye is released from the mitochondria when the mPTP are open. The change in mitochondrial fluorescence thus reflects the degree of mPTP opening. The fluorescence intensity of the mitochondria was determined using a microplate reader (TECAN Infinite M1000, Austria) at an excitation wavelength of 488 nm and an emission wavelength of 505 nm. The data were normalized to the control fluorescence.

### Determination of Mitochondrial Reactive Oxygen Species (ROS) Production

The production of mitochondrial ROS was measured using the highly sensitive Genmed mitochondrial ROS fluorescence detection kit (Genmed Scientifics INC., Shanghai, China). Isolated mitochondria were stained with 5-(and-6)-chloromethyl-2′, 7′-dichlorodihydrofluorescein diacetate acetyl ester (CM-H2DCFDA). CM-H2DCFDA (excitation/emission: 490/530 nm) is a cell-permeable indicator of ROS, which remains non-fluorescent until the acetate groups are removed by intracellular esterases and oxidation occurs. Thus, the levels of ROS were determined by changes in the DCF fluorescence intensity using a microplate reader (TECAN Infinite M1000, Austria).

### Western Blot Analysis

The method was described in previous publication (Sun et al., 2011). Finally, the blots were visualized by enhanced chemiluminescence using a BioRad imaging system (Bio-Rad, Hercules, CA, United States). Densitometric analysis of the bands was performed using Gel Pro software (Media Cybernetics, Rockville, MD, United States).

### Statistical Analysis

Data were expressed as means ± SD from at least three independent experiments. Statistical comparisons between different groups were determined by one-way ANOVA or the Student–Newman–Keuls method with GraphPad Prism 5.0 software (SPAA Inc., Chicago, IL, United States). *P*-value < 0.05 was considered statistically significant.

## Results

### ATO Induces Mitochondrial Damage in H9c2 Myocardial Cells

**FIGURE 2:**
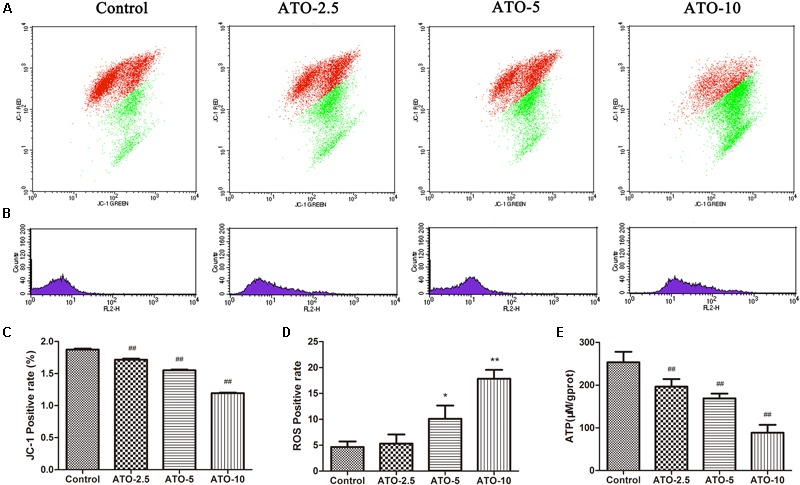
The effects of ATO treatment on mitochondrial injury in H9c2 cells. **(A)** A flow cytometric analysis of the mitochondrial membrane potential by JC-1 staining of H9c2 cells. **(B)** The effects of ATO on the mitochondrial ROS levels. **(C,D)** A statistical analysis of the flow cytometry data. **(E)** The effects of ATO on the ATP levels. The data are expressed as the means ± SD from three independent experiments. ^∗^*p* < 0.05 versus control; ^∗∗^*p* < 0.01 versus control.

Mitochondrial membrane potential is a prerequisite for the maintenance of mitochondrial function. We used JC-1 staining to detect changes in the myocardial mitochondrial membrane potential. The experimental results in **Figures 2A,C** show that compared with the control group, the proportions of red and green fluorescence intensity in the different dose groups of ATO increase, indicating that ATO treatment can cause the depolarization of the mitochondrial membrane potential in a dose-dependent manner. The mitochondria are the main sites for the production of ROS, and overproduction of ROS is an important mechanism for the induction of cardiomyocyte apoptosis. The flow cytometric results (**Figures 2B,D**) showed that ATO treatment can significantly increase the ROS content in the mitochondria. ATP is an indispensable universal energy source in all living organisms for cell biosynthesis. It plays an extremely important role in the metabolism of the body cells and provides energy for various biochemical reactions. The experimental results (**Figure 2E**) show that the ATO treatment group dose-dependently experienced a significant decrease in the ATP content.

### ATO Induces Cardiac Mitochondrial Damage in Mice

**FIGURE 3:**
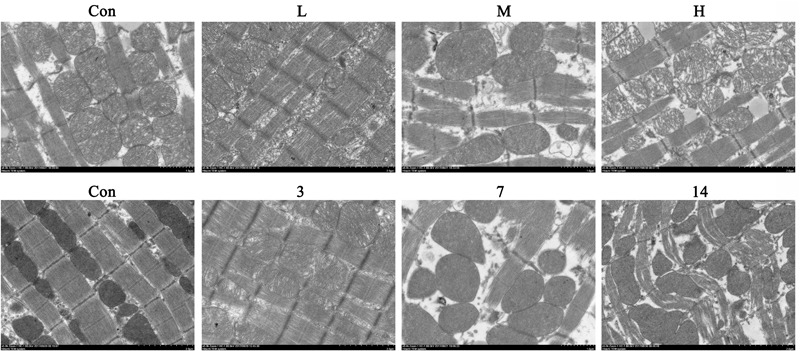
The ultrastructure of cardiomyocytes was observed using an electron microscope.

We observed the myocardial ultrastructure by scanning electron microscopy to assess the ATO-induced myocardial mitochondrial damage. No obvious morphological abnormalities were found in the mitochondria of normal mice. However, there were different degrees of mitochondrial damage in the H9c2 cells in the ATO treatment groups, which mainly consisted of the rupture of the outer membrane, the disappearance of swelling ridges, and the accumulation of mitochondria in some areas (**Figure 3**).

**FIGURE 4:**
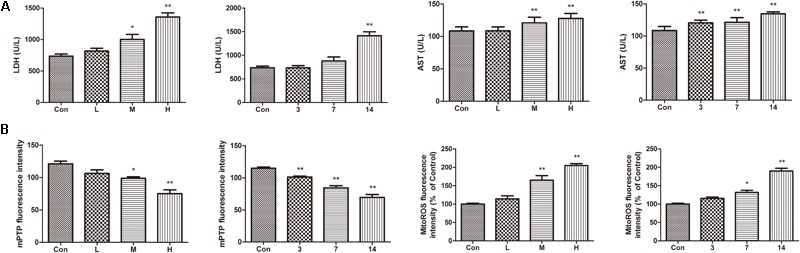
Effects of ATO on mitochondria injury in mouse heart. **(A)** Effects of ATO on the activities of LDH, AST in plasma; **(B)** The mPTP opening and mitochondrial ROS production in the cardiac mitochondria. The data are expressed as the means ± SD from three independent experiments. ^∗^*p* < 0.05 versus control; ^∗∗^*p* < 0.01 versus control.

Compared with the control group, the levels of AST and LDH in the serum of the ATO groups were significantly increased, indicating that ATO caused damage to the hearts of the mice in a dose-dependent manner (**Figure 4A**).

The continued opening of mitochondrial membrane pores (mPTP) is a major determinant of cell death. The opening of the mPTPs is closely related to the release of cytochrome c, the mitochondrial translocation of Bax and the increase of ROS levels. The open state of the mPTPs can be determined by changes in the mitochondrial calcein fluorescence. The average fluorescence intensity of calcein was significantly decreased in cardiomyocytes exposed to ATO treatment, suggesting that the mPTP opening was significantly increased (**Figure 4B**). In addition, ATO also caused a significant increase in the mitochondrial ROS levels in the myocardium.

### Effect of ATO on ATP Content in Mice

**FIGURE 5:**
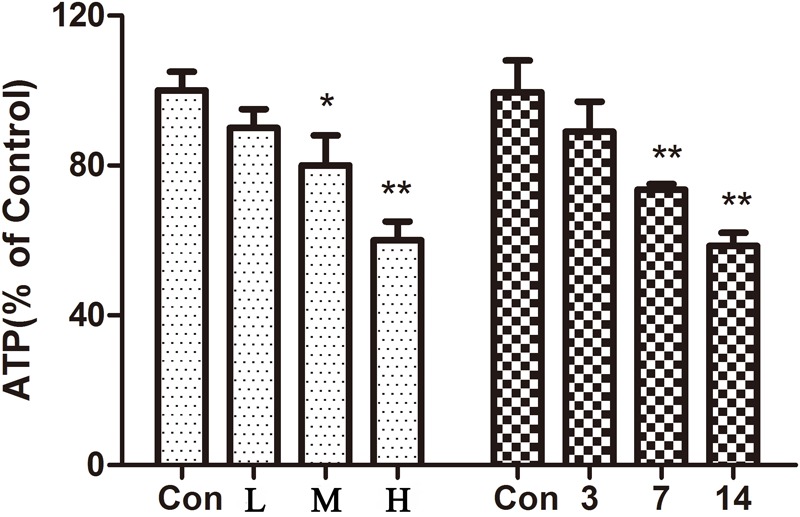
Effect of ATO on ATP in mice. The data are expressed as the means ± SD from three independent experiments. ^∗^*p* < 0.05 versus control; ^∗∗^*p* < 0.01 versus control.

The results showed (**Figure 5**) that compared with the control group, the ATP content in the hearts of mice gradually decreased with an increase in the administration time and the dose of ATO. ATO destroyed the mitochondrial energy supply to the mouse heart.

### Effects of ATO on Myocardial Mitochondrial Biogenesis

**FIGURE 6:**
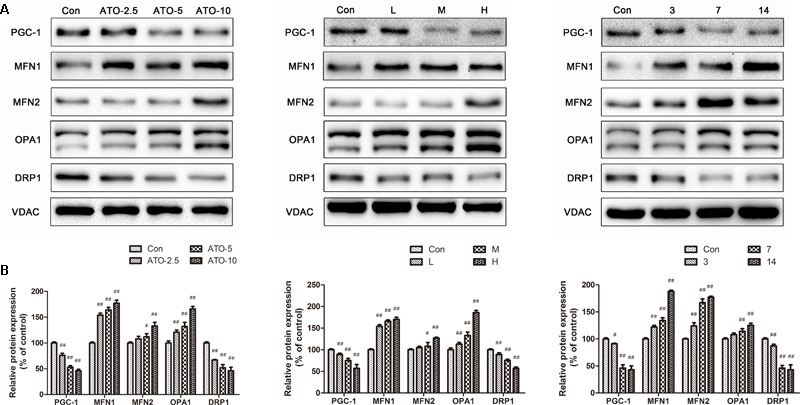
Effects of ATO on the expression levels of PGC-1α, MFN2, MFN, OPA1 and DRP1. Expression was assayed via a western blot analysis with a Gel-pro analyzer. The data are presented as the mean ± SD of three independent experiments. ^#^*p* < 0.05 versus control, ^##^*p* < 0.01 versus control.

PGC-1 plays a pivotal role in the regulation of mitochondrial biogenesis and metabolism in the heart. The results (**Figure 6**) showed a significant decrease of the biogenesis factor PGC-1α in the ATO treated group *in vitro* and *in vivo*. Furthermore, the protein expression of mitochondrial fusion and fission was also determined to discover their roles in mitochondrial dysfunction. Compared with the control group, the levels of DRP1 decreased significantly in the ATO groups, while the expression of MFN1, MFN2 and OPA1 increased significantly, indicating that ATO *in vitro* and *in vivo* affected the normal biogenesis and fusion of the myocardial mitochondria.

### Sal A Mitigates Myocardial Mitochondrial Damage Caused by ATO

**FIGURE 7:**
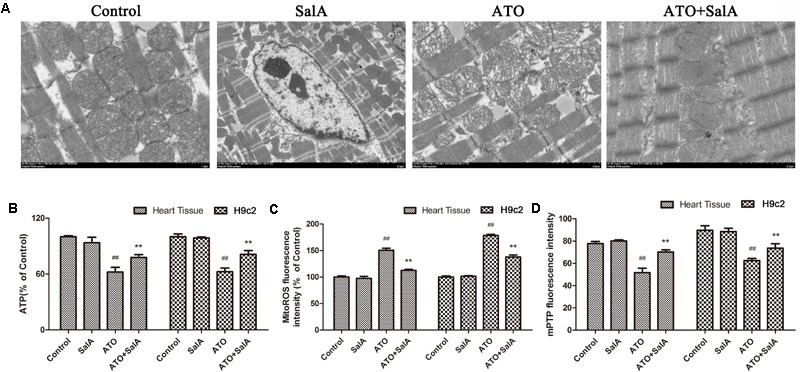
Effects of Sal A and ATO on mitochondrial injury *in vivo* and *in vitro*. **(A)** The ultrastructure of the cardiomyocytes was observed using an electron microscope. **(B)** ATP levels. **(C)** Mitochondrial ROS production in cardiac mitochondria. **(D)** Mitochondrial ROS production in cardiac mitochondria. All data are expressed as the mean ± SD of three independent experiments; ^##^*p* < 0.01 versus control, ^∗∗^*p* < 0.01 versus ATO.

We observed the ultrastructure of the cardiomyocytes by scanning electron microscopy to assess the ATO-induced cardiotoxicity. No obvious morphological abnormalities were found in the mitochondria of the myocardial cells in the normal group and the Sal A monotherapy group. However, mitochondrial swelling and mitochondrial membrane rupture occurred in cardiomyocytes of the ATO model mice. Sal A pretreatment significantly reduced these pathological changes (**Figure 7A**). The ATP levels were significantly decreased after ATO treatment compared to the control group. In contrast, pretreatment with Sal A significantly increased the ATP levels relative to the ATO group (**Figure 7B**). Opening of the mPTPs is an indicator of apoptosis, but it also affects the generation of energy. The experimental results in **Figures 7C,D** show that ATO significantly decreased the mitochondrial membrane potential and stimulated the production of mitochondrial ROS. However, Sal A pretreatment repairs the mitochondrial dysfunction, decreased the ATO-induced oxidative stress and maintained the mitochondrial membrane stability.

### Effect of Sal A on Myocardial Mitochondria Biogenesis

**FIGURE 8:**
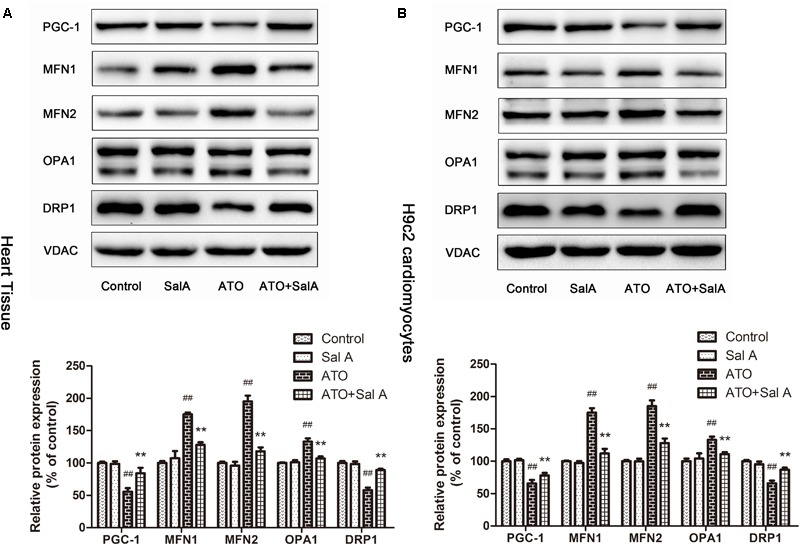
The effect of Sal A on mitochondrial biogenesis-related protein expression after ATO treatment. **(A)** Protein expression in the heart tissue. **(B)** Protein expression in H9c2 cells. All data are expressed as the mean ± SD of three independent experiments; ^##^*p* < 0.01 versus control, ^∗∗^*p* < 0.01 versus ATO.

PGC-1 is considered to be a major regulator of mitochondrial biogenesis, and the reduction of its level has been demonstrated in mitochondrial dysfunction. The experimental results (**Figure 8**) show that Sal A increased the downregulation of PGC-1 expression caused by ATO. Mitochondria continuously undergo changes in morphology and size through processes termed fission and fusion. ATO induced the abnormal expression of mitochondrial fission and fusion-related proteins. However, Sal A pretreatment can improve this trend by improving myocardial mitochondrial biogenesis.

### Sal A Enhances the Anticancer Capacity of ATO

**FIGURE 9:**
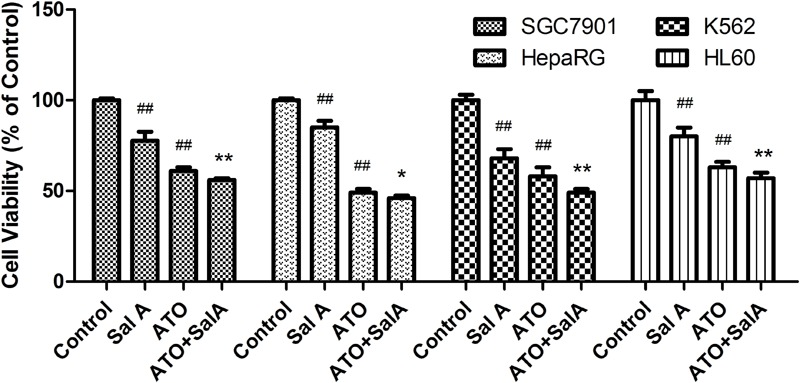
Effects of Sal A and ATO treatment on the cell viability of cancer cells. SGC7901, HepaRG, K562 and HL60 cells were treated with ATO (5 μM) in the presence or absence of Sal A (25 μM) for 24 h. Cell viability was determined by the CCK8 assay and expressed as a percentage of the control for each cell line. The data are expressed as the means ± SD of three independent experiments. ^##^*p* < 0.01 versus control; ^##^*p* < 0.01 versus control; ^∗^*p* < 0.05 versus ATO-treated cells; ^∗∗^*p* < 0.01 versus ATO-treated cells.

As shown in **Figure 9**, ATO showed a significant inhibitory effect on the activity of SGC7901, HepaRG, K562 and HL60 cells. Compared with the ATO model group, the Sal A and ATO combination treatment had a stronger inhibitory effect on the growth of these cancer cells. This finding indicates that the combination treatment may have the potential to enhance the antitumor effects of ATO.

## Discussion

Arsenic and its various forms have been used in ancient Chinese medicine for more than 2,000 years (Waxman and Anderson, 2001). Arsenic plays an important role in the treatment of syphilis and of various diseases such as cancer, which emphasizes its role as a therapeutic drug even if it is labeled a potential “poison” (Hoonjan et al., 2018). The ability of arsenic (especially ATO) to treat APL has fundamentally changed the understanding of this toxin and has been a major factor in the reappearance of this substance as a candidate in Western medicine for leukemia and other solid tumors (Kumana et al., 2002). The therapeutic use of arsenic is limited due to its severe cardiovascular side effects (Varghese et al., 2017). Mitochondria are important organelles in high-energy-consuming cardiomyocytes, which account for approximately 30% of the total cell volume (Detmer and Chan, 2007). Not only are they the primary source of ATP, but they are also involved in a large number of signaling cascades, which in turn regulate cell survival and cell death (Rosca et al., 2008). ATO-induced mitochondrial energy metabolism disorders, ROS production, an imbalance of calcium homeostasis, and an increase in intima-media permeability are important causes of cardiotoxicity (Zhao et al., 2008).

Over the past two decades, researchers have gradually focused on the protective effects of traditional Chinese medicines and natural medicines on myocardial mitochondrial damage (Camara et al., 2010). Many traditional Chinese medicines and natural medicines have been shown to have a definite curative effect and few adverse reactions when used for the prevention and treatment of heart-related diseases (Li et al., 2009; Wu, 2009). Therefore, with increased research and further clarification of the damage mechanism of mitochondria focused on the direct or indirect protection of mitochondrial integrity, the maintenance and control of mitochondrial homeostasis, and the enhancement of the tolerance of mitochondria to natural drugs will become important in the prevention and treatment of cardiac toxicity using an ATO strategy (Bhattacharya, 2017).

In this study, a dose-response study revealed that ATO treatment led to a series of changes in the structure and function of the mitochondria, including impaired ATP synthesis, increased opening of the mPTP, and especially a burst of ROS generation, which resulted in metabolic disorders and oxidative damage. However, Sal A pretreatment significantly increased the intracellular ATP levels, reduced the mitochondrial ROS production, reduced the mPTP opening, and maintained the mitochondrial membrane potential, which suggested that Sal A can improve myocardial cell mitochondrial function and energy status after an ATO injury. This is consistent with other results, indicating that Sal A has a significant mitochondrial protective effect on H/R injury.

Mitochondrial biogenesis comprises the growth and division of pre-existing mitochondria. PGC-1 is highly expressed in cardiac myocytes and has been confirmed to be key factor for myocardial mitochondrial biogenesis (Di et al., 2018). PGC-1 has been reported to play a critical role in cardioprotective therapies (Lehman et al., 2000). In our study, ATO significantly decreased the protein expression of PGC-1, suggesting that ATO markedly suppressed mitochondrial biogenesis. We also observed that the expression level of PGC-1 was significantly increased in Sal A-pretreated group compared with the ATO group. The mitochondrial division and fusion processes are precisely regulated by a variety of proteins (Kasahara et al., 2013). MFN1 and MFN2 are key proteins involved in mitochondrial outer membrane fusion. The fusion of the mitochondrial inner membrane is mainly mediated by OPA, and DRP1 and FIS1 are involved in the regulation of mitochondrial division (Chan, 2006; Westermann, 2008). In this experiment, ATO treatment decreased the expression of the mitochondrial division protein Drp1 and increased the expression of the mitochondrial fusion proteins Opa1, Mfn1 and Mfn2, indicating that ATO inhibits normal mitochondrial division and promotes a detrimental increase in mitochondrial fusion. In contrast, Sal A pretreatment can increase the expression of the mitochondrial protein Drp1 and decrease the expression of mitochondrial fusion protein, indicating that Sal A treatment can maintain the normal mitochondrial division and fusion state. It has been suggested that the cardiac protective effect of Sal A is related to its ability to maintain the integrity of mitochondria by regulating mitochondrial fusion and division.

Although our previous research and the current data showed that Sal A can attenuate ATO-induced cardiotoxicity *in vitro* (Zhang et al., 2017). Another important question is whether Sal A reduces the anti-cancer activity of ATO. Recent studies have demonstrated that Salvianolic acid A reverses cisplatin resistance in lung cancer A549 cells (Tang et al., 2017). The component formula of Salvia miltiorrhiza and Panax ginseng induces apoptosis and inhibits cell invasion and migration by targeting PTEN in lung cancer cells (Bi et al., 2017). In our study, we found that Sal A enhanced the antitumor activity of ATO in SGC7901, HepaRG, K562 and HL60 cancer cells. All these results suggest that Sal A has the potential to be an adjuvant for future clinical applications.

Nevertheless, this study is in its initial stages. The results of the anti-cancer activity *in vitro* experiments still need to be verified in animal models. Our data do not fully explain the direct relationship between mitochondrial protection and related signal pathways. It’s reported that Sal A could protect cardiomyocytes against Hypoxia/Reoxygenation-induced injury by preserving mitochondrial function and activating Akt/GSK-3β signals (Li et al., 2017). The suppression of GSK-3 activity by Akt-mediated Ser9 phosphorylation in the mitochondria affords cardiomyocytes tolerance against apoptosis. The above information all indicate that Akt/GSK-3β signals may play an important role in preserving mitochondrial function. Therefore, a more thorough investigation is necessary to further explore the mechanisms of Sal A.

## Conclusion

In conclusion, ATO induces cardiac mitochondrial damage, including structural damage, abnormal mPTP opening, massive production of ROS, impaired energy metabolism, and downregulation of mitochondrial biogenesis. Accumulation of these maladjusted characteristics triggers a vicious circle, further exacerbating the cardiotoxicity of ATO. Sal A is one of the main active ingredients of traditional Chinese medicine *Salvia miltiorrhiza Bunge*. It improves ATO-induced cardiotoxicity by improving mitochondrial structure and function and upregulating mitochondrial biosynthesis. In addition, Sal A also enhances the antitumor activity of ATO *in vitro*, indicating that the combination of Sal A with ATO has potential clinical application.

## Author Contributions

X-bS, G-bS, and J-xY participated in the research design. J-yZ, MW, and R-yW conducted the experiments and data analysis. XS and Y-yD contributed to the revision of the manuscript.

## Conflict of Interest Statement

The authors declare that the research was conducted in the absence of any commercial or financial relationships that could be construed as a potential conflict of interest.
